# Allopregnanolone Is Associated with a Stress-Induced Reduction of Heart Rate Variability in Premenstrual Dysphoric Disorder

**DOI:** 10.3390/jcm12041553

**Published:** 2023-02-16

**Authors:** Ajna Hamidovic, John Davis, Fatimata Soumare, Aamina Naveed, Yaseen Ghani, Selma Semiz, Dina Khalil, Margaret Wardle

**Affiliations:** 1 Department of Pharmacy, University of Illinois at Chicago, 833 S. Wood St., Chicago, IL 60612, USA; 2 Department of Psychiatry, University of Illinois at Chicago, 1601 W. Taylor St., Chicago, IL 60612, USA; 3 Shirley Ryan Ability Lab, 355 East Erie Street, Chicago, IL 60611, USA; 4 Department of Psychology, Leiden University, Rapenburg 70, 2311 EZ Leiden, The Netherlands; 5 Education Development Center, 300 Fifth Avenue, Suite 2010, Waltham, MA 02451, USA; 6 Department of Psychology, University of Illinois at Chicago, 1007 W. Harrison St., 1009 BSB, Chicago, IL 60607, USA

**Keywords:** premenstrual dysphoric disorder (PMDD), stress, heart rate variability (HRV), allopregnanolone, Trier Social Stress Test (TSST), parasympathetic nervous system, ultra-performance liquid chromatography tandem mass spectrometry

## Abstract

Human survival and wellbeing require appropriate responses to stress, including a highly coordinated and efficient nervous system control of the heart rhythm. During stress, a greater disinhibition of the vagal nerve is reflective of poor stress adaptability, which may be relevant in premenstrual dysphoric disorder (PMDD)—a debilitating affective condition thought to be marked by dysregulated stress processing and sensitivity to allopregnanolone. In the present study, women with PMDD (n = 17) and healthy controls (n = 18), who did not take medication, smoke, or consume illicit drugs, and who were free of other psychiatric conditions, participated in the Trier Social Stress Test, during which we measured the high frequency of the heart rate (HF-HRV) and allopregnanolone using ultra-performance liquid chromatography tandem mass spectrometry. Relative to their baseline, women who have PMDD, but not the healthy controls, experienced a reduction in HF-HRV during stress anticipation (*p* ≤ 0.05) and stress (*p* ≤ 0.01). Their recovery from stress was significantly delayed (*p* ≤ 0.05). Absolute peak HF-HRV change from baseline was significantly predicted by baseline allopregnanolone only in the PMDD group (*p* ≤ 0.01). The present study shows how an interaction between stress and allopregnanolone—which have both been separately implicated in PMDD—underlies PMDD expression.

## 1. Introduction

Premenstrual dysphoric disorder (PMDD) is a psychiatric condition in which women experience a cyclical emergence of emotional and physical symptoms in the luteal phase of the menstrual cycle with remission in the week following menses [1]. Across roughly 450 menstrual cycles that a woman has in her lifetime, a PMDD patient who experiences symptoms for one week per cycle would experience approximately 8.5 cumulative years of symptoms—similar to what a patient with recurrent major depressive disorder (MDD) would experience across his/her lifetime [2,3,4]. The disorder affects between 5–7% of reproductive age women [4,5,6], and, similar to MDD, has an estimated morbidity of 14.5 million disability-adjusted life-years in the United States [2].

There is a critical need for an improved understanding of PMDD etiology, which appears to be multifactorial, and includes a noted involvement of dysregulated stress processing [7,8]. Women suffering from severe premenstrual symptoms self-report higher perceived chronic stress [9]. Elevated daily hassles score and number of traumatic events are significant risk factors for developing PMDD, as shown in a prospective, longitudinal study involving 1, 488 women [10]. Women with PMDD have a delayed cortisol awakening response peak and a flattened diurnal cortisol slope [11], which seemingly reflect a trait, and not an effect of menstrual cycle phase [11].

Little is known about stress-related cardiovascular function in women with PMDD, despite the fact that heart disease is the leading cause of death for women in the United States [12]. A particularly important marker of stress effect on the heart is heart rate variability (HRV), which is the inter-beat interval variation between consecutive heartbeats. HRV is an autonomic reflection of emotional regulatory abilities to flexibly respond to challenges, such as psychosocial stress (reviewed in Balzarotti et al. [13]).

The heart is dually innervated by the autonomic nervous system such that relative increases in sympathetic and parasympathetic activities result in heart rate increases and decreases, respectively. Under resting conditions, parasympathetic (vagal) activity dominates, while sympathetic activation and parasympathetic withdrawal occur in response to stress. Greater parasympathetic withdrawal is reflective of poor adaptability to stress; a balanced system of high variability in the heart rate is healthy because the system can effectively respond to physical and environmental demands. A system that is “locked in” to a particular pattern is considered dysregulated. Therefore, the heart rate of a healthy heart oscillates spontaneously (i.e., shows high HRV), whereas the heart which shows low variability is reflective of poor health [14].

Our recent analysis showed a significant effect of sex on the ability to regulate HRV during stress. Analyzing data from 17 studies, constituting 929 individuals who underwent the Trier Social Stress Test (TSST), we found lower HRV in women during task delivery, reflecting a worse control of the parasympathetic tone. We found no group differences at baseline, and the differences during anticipation and recovery were marginal, with women, again, demonstrating lower HRV. Different methods of measuring HRV have been developed. Time (for example, the root mean square of successive differences between normal heartbeats (RMSSD) and frequency domain analyses at high frequency (HF-HRV; 0.15–0.40 Hz)) reflect parasympathetic activity. Measurement of sympathetic activity using heart rate measurements (i.e., low frequency HRV; LF-HRV) is controversial [15]; hence, unless specified as LF-HRV, HRV generally refers to the measurement of the parasympathetic tone. Our meta-analytic approach was designed to specifically evaluate the parasympathetic activity; therefore, the finding of lower HRV during stress in women reflects a reduced parasympathetic tone.

The noted sex difference in HRV regulation during stress [16] suggests that sex hormones and/or their potent metabolites mediate stress-induced HRV changes. In particular, the activity of 3α-hydroxy-5α-pregnan-20-one, or allopregnanolone—a metabolite of progesterone with potent GABAA receptor-modulating effects—has been implicated in the etiology of PMDD. In a randomized, double-blind, placebo-controlled crossover study conducted by Martinez et al. [17], dutasteride, a 5-α reductase inhibitor (which blocks the conversion from progesterone to allopregnanolone), prevented symptom expression in women with PMDD. Indeed, both rodent and human studies provide evidence of impaired interactions between allopregnanolone and the GABAA receptor in PMDD, as reviewed by Hantsoo and Epperson [18]. Moreover, the neurotransmitter GABA can modulate HRV via central mechanisms [19] and muscimol, an ionotropic GABAA receptor agonist [20], reduces the high frequency spectral power of the heart rate during a restraint test [21].

As there are no studies to date examining autonomic function in women with PMDD during stress, we completed the proposed study to evaluate whether stress-related changes in HRV are distinctive in PMDD, hypothesizing a significant stress-induced reduction of HRV in women with PMDD relative to the healthy controls. Additionally, based on the previous findings showing allopregnanolone sensitivity in women with PMDD and the involvement of the GABAA receptor on the modulation of HRV during stress, we hypothesized that HRV changes during stress would be mediated by allopregnanolone in women with PMDD, but not the healthy controls.

## 2. Materials and Methods

### 2.1. Study Design

Premenstrual Hormonal and Affective State Evaluation (PHASE) is a single-cohort longitudinal design study with a nested human laboratory between subject experiment (i.e., TSST). The study enrolls women with regular menstrual cycles to chart their symptoms using Daily Record of Severity of Problems (DRSP) [22] and menstruation timing during two–three menstrual cycles. In the final menstrual cycle of the study, study participants complete: (1) blood and salivary sample collection at 8 different times of the menstrual cycle, (2) psychosocial stress testing in the luteal phase, and (3) urinary luteinizing hormone self-testing. Knowledge gained from PHASE is expected to increase our understanding of menstrual cycle physiology and its dysregulated states. PHASE is a registered clinicaltrials.gov study (NCT03862469). The University of Illinois Human Research Protection Office (HRPO) approved the consent form and all other documents for the study (HRPO ID: 2018-1533).

### 2.2. Study Participants

Women between the ages of 18 and 35, with regular menstrual cycles lasting 21 to 35 days [23,24,25] were recruited from the general population using flyers, word-of-mouth referrals, and electronic media (Facebook, Instagram, and Craigslist). The study was advertised as “what causes severe premenstrual syndrome (PMS)?” and the ad indicated that women with and without severe PMS may be eligible to participate. We used the term “severe PMS”, as most individuals are familiar with PMS, rather than PMDD (i.e., the severe form of PMS). For all study participants, diagnosis was re-established using the criteria discussed in the “Diagnosis” section.

Study participants first completed an online survey, following which they were scheduled to complete an in-person screening. The exclusion criteria were: (a) lifetime DSM-5 Axis I disorder, except anxiety and depression (based on the Structured Clinical Interview for DSM Disorders (SCID)), (b) current (i.e., within the past 12 months) DSM-5 Major Depressive Disorder or an anxiety disorder (based on SCID), (c) positive urine drug screen test, (d) breath alcohol concentration > 0.00%, (e) Alcohol Use Disorders Identification Test (AUDIT) score > 7, (e) self-reported smoker or carbon monoxide concentration ≥ 6 ppm, (f) irregular menstrual cycle, (g) current pregnancy (urine test-verified) or lactation, or a plan to become pregnant, (h) moderate or high suicide risk, (i) Shipley IQ (vocabulary standard score) < 80, (j) prescription medications, and (k) hormonal contraception.

### 2.3. Study Procedures

Once enrolled and having begun their subsequent menstrual cycle, study participants completed DRSP in Research Electronic Data Capture (REDCap) between 7 PM and midnight each day while in the study. In addition, study participants were scheduled to complete the Trier Social Stress Test in the mid/late luteal phase of the last menstrual cycle while in the study (i.e., second or third). The session started at 11:00 with urine drug screen collection for the purpose of ruling out drug intake, along with CO and alcohol breath testing for the purpose of ensuring absence of smoking or alcohol intake. An intravenous catheter placement procedure was completed at 11:10. Participants entered the relaxation phase from 11:20 to 12:40, during which they read neutral materials or watched a neutral movie. The relaxation phase was necessary for any stress encountered as the result of catheter insertion to subside. In addition, a continuous heart rate monitor, ZephyrTM BioHarness 3.0 (Zephyr Technology Corporation, Annapolis, MD, USA), was placed at 13:00 and removed at 14:10.

The Trier Social Stress Test [26] started at 13:30 and ended at 13:50. The task is described in detail in Childs and de Wit (2010). In summary, it involves preparation for the task (10 min), delivering the speech and math test (5 min each) in a different room in front of a panel of judges, and returning to the original room for collection of various samples. Blood samples were collected at −20, 0, +10, +20, +30, +45, and +60. The discharge procedure included removal of the intravenous line, completion of adverse event form, and debriefing.

### 2.4. Diagnosis

The Diagnostic and Statistical Manual of Mental Disorders 5 (DSM-5) specifies the possible symptoms of PMDD as: (1) affective lability (mood swings), (2) irritability or anger, (3) depressed mood, (4) anxiety or tension, (5) decreased interest in usual activities, (6) difficulty concentrating, (7) a sense of being overwhelmed or out of control, (8) change in appetite, overeating, or specific food cravings, (9) hypersomnia or insomnia, (10) fatigue, and (11) one physical symptom (for example, breast tenderness). PMDD diagnosis requires the presence of at least one affective symptom (symptoms 1–4) to reach the total of 5 required symptoms, which must be confirmed in a prospective manner for at least 2 menstrual cycles. In addition, the symptoms must be associated with clinically significant distress or interference with work, school, usual social activities, or relationship with others.

In accordance with DSM-5 criteria, PMDD diagnosis in the proposed study was be assessed prospectively by evaluating the participants’ daily symptom ratings using the DRSP scale [22] during two–three menstrual cycles. PMDD diagnosis was defined as a 30% or greater increase in 5 or more symptoms, one of which had to be affective, as well as functional impairment, between the luteal (day −7 to −1) and follicular (day 6 to 12) days relative to the range of the scale of each individual participant across the entire menstrual cycle [27]. PMDD participants defined using these criteria were found to have differential cellular [27,28] and sex hormone processing [29], as well as unique transcriptional responses [30].

Of note, and in references to the discussion section, the diagnosis of premenstrual syndrome (PMS) requires a prospective assessment of symptomatology, with the presence of 1 to 4 symptoms and without the need that one of them must be affective.

### 2.5. Measures

Heart Rate Variability. Trained research assistants edited the resulting interbeat intervals for irregular beats using CardioEdit software (Brain-Body Center, University of Illinois at Chicago, Chicago, IL, USA). HF-HRV was quantified from these interbeat interval sequences using CardioBatch software (Brain-Body Center) and the moving polynomial method [31] with standard adult HF-HRV settings: 2 Hz sample rate, frequency window of 0.12–0.40 Hz, and 30-s epoch length. The data were segmented into 30-s epochs and then averaged across those epochs in order to reduce distortion. The final values represent the average of the natural logarithm transformed variance of each epoch.

Allopregnanolone. The analysis of allopregnanolone was performed by the Mass Spectrometry Core in the Research Resources Center of the University of Illinois at Chicago. Allopregnanolone was dissolved in either methanol or acetonitrile to get a stock solution of 1 mg/mL. The stock solution was then diluted 1000 ng/mL, 750 ng/mL, 500 ng/mL, 200 ng/mL, 100 ng/mL, 50 ng/mL, 25 ng/mL, 10 ng/mL, 5 ng/mL, 1 ng/mL, 0.5 ng/mL, 0.1 ng/mL, and 0.05 ng/mL using 50% methanol in water as the spiking standards to prepare the standard curve. Internal standards were diluted to 1 ug/mL in 50% methanol as the working solution for standard curve/sample preparation. Calibrators used to construct the standard curve for positive mode analysis were prepared by taking 90 uL from each point of the standard spiking solution and drying it under a stream of nitrogen. Dried residue was dissolved in 50 mM sodium bicarbonate (45 uL, pH 10), derivatized by adding dansyl chloride (45 uL, 1 mg/mL in acetone) and incubating at 60 °C for 10 min. Internal standard was derivatized following the same procedure and used for spiking each point of the curve. Analyses were conducted using an AB SCIEX 6500 QTRAP mass spectrometer coupled with Agilent 1290 UPLC system. All samples were eluted from an Agilent Poroshell 120 SB-C18 2.7 µm column (2.1 × 100 mm) with a flow rate of 200 µL/min. The column compartment was kept at 50 °C. The gradient started with a 95% mobile phase A (0.1% formic acid in H_2_O) for 2 min followed by a linear gradient increase of the mobile phase B (0.1% formic acid in MeOH) from 5% to 80% in 2 min, 80% to 90% over 2 min, and kept at 90% of B for 7 min, and then re-equilibrated back to the initial condition (95% A) for 3 min, resulting in a total separation time of 16 min. Mass spectrometry experiments were performed via MRM scan using electrospray ionization (ESI) in positive ion mode with an ESI spray voltage of 4.5 kV and a source temperature of 500 °C. The limit of detection of allopregnanolone ranged from 5–25 pg. The lower limit of quantification of allopregnanolone ranged from 25–50 pg. Each calibration standard’s accuracy was within the acceptable range of 15%. The recovery of allopregnanolone was assessed for quality control samples at 3 levels (low, mid, high) during the initial method development. For allopregnanolone, recovery of 75 pg, 300 pg, and 600 pg were 99.8%, 101.1%, and 105.6%, respectively. Allopregnanolone and allopregnanolone-D4 internal standard were supplied by Steraloids (Newport, RI, USA). LCMS grade methanol, acetonitrile, acetone, and water were obtained from Optima, Fisher Scientific (Waltham, MA, USA). LCMS grade formic acid was obtained from Pierce, Thermo Scientific (Waltham, MA, USA). Tert-Butyl methyl ether and dansyl chloride were obtained from Sigma-Aldrich (St. Louis, MO, USA).

The Beck Depression Inventory (BDI) [32] is a 21-item, self-report rating inventory that measures characteristic attitudes and symptoms of depression. Internal consistency for the BDI ranges from 0.73 to 0.92, with a mean of 0.86 [33]. The BDI demonstrates high internal consistency, with alpha coefficients of 0.86 and 0.81 for psychiatric and non-psychiatric populations respectively [33]. Study participants completed the BDI at screening, and the purpose was to ensure that our procedure for screening out participants with current MDD was efficient.

### 2.6. Data Analyses

A baseline (i.e., resting) HF-HRV was collected from 13:05:00–13:10:00, while the participants sat with their eyes closed. The baseline timepoint was defined as a 3-min period from the middle of the baseline recording, and 90 s out each way. The instruction HRV timepoint was measured from the start of the instruction (13:30:00, or as written on the flowsheet) until +3 min. For the TSST timepoint, we analyzed HRV during a 3-min period from the start of the math test (13:45:00, or as written on the flowsheet) + 3 min. Finally, we defined recovery from 10 s after the participant exited the TSST speech room (approximate time to reach the data collection room) (13:50:10, or as written on the flowsheet) + 3 min.

### 2.7. Statistical Analyses

All statistical analyses were performed in R software (version 4.0.2) [34]. We compared the demographic characteristics of the study groups using the Chi Square (or Fisher Exact) test for categorical, and the analysis of variance (ANOVA) for continuous, variables. Distribution of all variables was tested using the Shapiro–Wilk normality test, and, if not normally distributed, data were transformed using the square root function. We then used linear mixed-effects (LME) models using the R package *nlme* to study the differences in HF-HRV according to the group. Specifically, the model included the 4 timepoint (factor) variable, the 2 group (factor) variable, and their interaction term as fixed effects and a random intercept within each subject. All analyses were adjusted by the Tukey method for comparing multiple estimates. We analyzed allopregnanolone with the model that included the 7 timepoint (factor) variable, the 2 group (factor) variable, and their interaction term as fixed effects, and a random intercept within each subject, with the Tukey adjustment. We defined the statistical significance as an adjusted *p*-value < 0.05.

We next evaluated baseline allopregnanolone as a predictor of HF-HRV. We focused on baseline allopregnanolone because there were no changes in allopregnanolone as a result of the TSST procedure. To summarize HRV trajectory, we defined the peak change from baseline as HRV_baseline_-HRV_peak_ and the return from peak as HRV_recovery_-HRV_peak_. In these analyses, the peak was defined as the absolute value of the nadir because HRV is expected to decrease as a result of stress. We ran two linear regression models; the first predicting HRV peak change from baseline as an interaction between baseline allopregnanolone and diagnosis, and the second HRV return from peak as an interaction between baseline allopregnanolone and diagnosis.

## 3. Results

### 3.1. Study Participants

The present analysis included 35 participants: 17 with PMDD and 18 healthy controls. Overall, the participants were approximately 26 years old (26.25 ± 5.18 (mean ± SD)). They were mostly White (n = 14) and Asian (n = 12). Their average age of menarche was approximately 12 years old (11.89 (1.61) (mean ± SD)). Beck’s Depression Inventory mean score was 2.97, with SD of 2.83, indicative of an appropriate method to exclude participants with major depressive disorder using SCID (as listed in Section 2.2). Table 1 lists demographic characteristics of study participants, with *p* values showing no differences between the study groups on basic demographic measures. TSST sessions in the present study were completed in the mid or late luteal subphases of the menstrual cycle. Post-hoc analyses of luteinizing hormone, progesterone levels, and menstrual cycle duration (as described in Hamidovic et al. [35])confirmed the mid and late luteal subphases in 31 study participants. Of those, nine women (five with PMDD and four healthy controls) completed the TSST in the mid-luteal subphase. A total of two participants, though, had already started their next menstrual cycle (both were tested on day 1 of their subsequent menstrual cycle), while four participants’ cycles were anovulatory. Hence, 83% of the participants ovulated and were tested in the mid or late luteal subphases of the menstrual cycle. We first completed all analyses including the total study sample (n = 35), and then again completed the analyses excluding the aforementioned six study participants (four who did not ovulate and two who completed their testing on the first day of their subsequent menstrual cycle). The results remained similar whether the full or the partial sample was analyzed; hence, we present the results for the full sample in the main text and the results of the partial sample (for any significant findings in the full sample) in the supplementary materials.

### 3.2. High Frequency Heart Rate Variability

The Shapiro–Wilk normality test was not significant for the HF-HRV data at any of the four timepoints; hence, the data were analyzed in their original form. We first ensured that HF-HRV changed across the timepoints as a result of stress in the overall group. As expected, we observed significant changes across the timepoints, with an expected U shape trajectory and the most significant decrease in HF-HRV during speech (Supplementary Table S1—full sample; Supplementary Table S2—partial sample).

**Figure 1:**
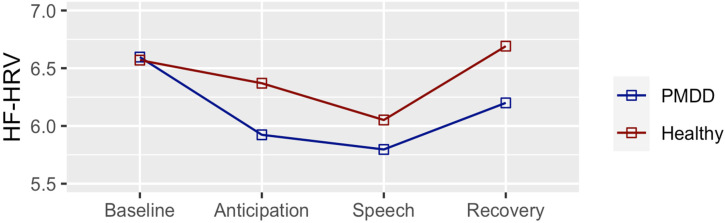
HF-HRV Changes Across Different Phases of the Trier Social Stress Test According to Group. Relative to baseline, HF-HRV of the PMDD group was reduced during anticipation (*p* ≤ 0.05) and speech (*p* ≤ 0.01). No significant changes relative to baseline were observed in the healthy participants, who displayed a significant difference in the speech vs. recovery contrast (*p* ≤ 0.05). This contrast was not significant in the PMDD group.

We next examined whether these trajectories varied according to group. As shown in Figure 1 and Table 2, HF-HRV of the PMDD group was already reduced (relative to baseline) during the instruction (i.e., anticipation) period (estimate = −0.67; SE = ±0.24; *p* ≤ 0.05), while this contrast remained unchanged in the healthy control group. The reduction in HF-HRV (relative to baseline) was even more significant during TSST speech delivery in the PMDD group (estimate = −0.8; SE = ±0.24; *p* ≤ 0.01), while, again, it was not significant in the healthy control group. The healthy control group recovery (TSST vs recovery) was significant (estimate = 0.40; SE = ±0.24; *p* ≤ 0.05), while it was not significant in the PMDD group. The comparisons between groups at each timepoint were not significant. Supplementary Table S3 shows means, SD, and SEM of HRV values according to groups at the four different timepoints and Supplementary Table S4 shows the results of this analysis in the partial sample.

### 3.3. Allopregnanolone

Study nurses were not able to draw blood samples from three study participants; hence, allopregnanolone values were available for 32 participants. The Shapiro–Wilk normality test was significant at each of the seven timepoints and the data were transformed using the square root method, which normalized the distribution. Baseline allopregnanolone (i.e., pre-stress) did not differ between the mid-luteal and late luteal subphases of the menstrual cycle. Allopregnanolone did not change significantly across time in the overall group, or according to diagnosis. We show means, SD, and SEM of HRV values according to groups at the seven different timepoints in Supplementary Table S5.

### 3.4. Association between Allopregnanolone and HRV

**Figure 2:**
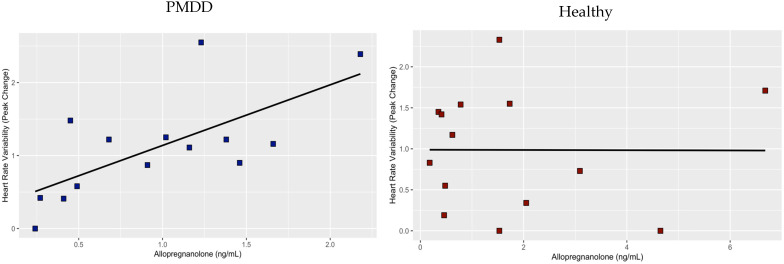
Association between HRV as peak change form baseline and baseline allopregnanolone in PMDD (**left**) and healthy (**right**) study participants. Whereas the association is significant in the PMDD group (*p* ≤ 0.01), this association was not identified in the healthy control group.

All data were square root transformed for the two linear regression analyses. The first linear regression analysis, examining HRV peak change from baseline predicted by an interaction between diagnosis and baseline allopregnanolone levels, showed that HF-HRV is significantly predicted from allopregnanolone (estimate = 1.47; SE = 0.55; *p* = 0.01), diagnosis (estimate = 0.45; SE = 0.21; *p* = 0.04, and allopregnanolone*diagnosis (estimate = −0.52; SE = 0.20; *p* = 0.01). We plotted and analyzed linear regression separately for each of the groups, showing distinct associations. While the association between allopregnanolone and peak change from baseline was significant in the PMDD group (estimate = 0.94; SE = 0.26; *p* = 0.003) (Figure 2, left), this association was not significant for the healthy control group (Figure 2, right). In the partial sample, examining HRV peak change from baseline predicted by an interaction between diagnosis and baseline allopregnanolone levels, showed that HRV is significantly predicted from allopregnanolone (estimate = 1.66; SE = 0.62; *p* = 0.01), diagnosis (estimate = 0.47; SE = 0.22; *p* = 0.04), and allopregnanolone*diagnosis (estimate = −0.59; SE = 0.22; *p* = 0.01). The association between allopregnanolone and peak change from baseline was significant in the PMDD group (estimate = 1.06; SE = 0.31; *p* = 0.008), this association was not significant for the healthy control group. The linear regression examining HRV return from baseline did not show that baseline allopregnanolone, diagnosis, or the interaction between allopregnanolone and diagnosis are significant predictors.

## 4. Discussion

The results of the present study demonstrate a distinct time course of HF-HRV in response to stress in women with PMDD. Relative to their baseline HF-HRV, women who have PMDD experienced a reduction in HF-HRV during stress anticipation, and a further reduction during stress. Moreover, the recovery from stress was delayed in women with PMDD. Hence, the present results further support the notion that PMDD is a psychiatric condition marked by an underlying dysregulation of stress processing. Finally, our study demonstrates that stress-induced HF-HRV reduction in women with PMDD is mediated by their baseline allopregnanolone level, showing how an interaction between the autonomic control of heart rate during stress and allopregnanolone underlies PMDD expression.

HRV was examined in several studies to date in women who experience PMDD and premenstrual syndrome PMS—the less severe form of PMDD. Evaluating HF-HRV during sleep in 9 women with PMDD and 12 healthy controls, Baker et al. [36] found lower HF-HRV in the PMDD group during non-REM sleep, but similar values during the REM stage. These findings, however, were not replicated in a study by de Zambotti et al. [37], though progesterone levels correlated positively with HF power in control women in NREM sleep. Comparing time and frequency domain variables in 28 women with PMDD and 11 healthy controls during rest, Landen et al. [38] found similar time (rMSSD and SDNN) and frequency (HF) domain measures of vagal activity in the luteal phase of the menstrual cycle. This result is consistent with the findings from the present study, which did not show group differences in the baseline HF-HRV. Rather, the differences in the present study emerged as a result of different HR-HRV trajectories across psychosocial stress phases. The results of Matsumoto et al. [39], however, showed that resting HF-HRV is decreased in women with PMDD (relative to the healthy controls as well as women with PMS) in the luteal phase of the menstrual cycle. Luteal and follicular phases were determined using the calendar method, without control of ovulation. Therefore, further studies examining fine-grained subphase (early follicular, mid-follicular, periovulatory, early luteal, mid-luteal, and late luteal) contrasts, and using serum LH values to properly align menstrual cycle subphases [40], will be valuable in examining whether resting HRV differentially changes across the entire menstrual cycle in PMDD relative to healthy controls.

Two studies to date examined changes in HRV in response to stress [41,42] in women with PMS vs healthy controls. Meng et al. [41] examined HF-HRV changes as a result of social stress; however, no effect of stress on HF-HRV was captured. Moreover, no effect of group, analyzed as a change from baseline for PMS vs healthy control, on HF-HRV was observed. Similarly, social stress procedure in Oda et al. [42] did not reduce HR-HRV, and the comparison of HF-HRV trajectories did not show a significant difference between the groups. In addition to the problem related to the absence of stress effects on HR-HRV in these two studies, the PMS diagnosis was assigned by retrospectively collecting menstrual cycle symptom data, which is in conflict with the DSM-5 method of assigning PMS diagnosis, requiring prospectively assessed presence of 1–4 premenstrual symptoms, without the requirement that one must be affective in nature. Hence, the present study improves the study of autonomic control in women with PMDD during stress by prospectively assigning PMDD diagnosis, ensuring presence of affective symptomatology, and 5 or more premenstrual symptoms, as required in DSM-5, as well as properly inducting stress effects on HF-HRV (Supplementary Tables S1 and S2).

Higher concentrations of allopregnanolone in PMDD participants, but not the control group, were associated with greater HF-HRV stress-induced reductions in the present study. Like the related positive GABAA receptor modulators benzodiazepines, alcohol, and barbiturates, allopregnanolone is anxiolytic, and it normally induces sedation and calmness. However, in some individuals, all GABAA receptor modulators can have paradoxical anxiogenic effects. This effect is biphasic—anxiogenic effects are observed at low levels (including luteal phase allopregnanolone concentrations), but with further concentration increases, there is a decrease in symptom severity [43,44,45,46,47]. Concentration, however, is one, but seemingly not the only, determinant of this effect [43,46]. Based on the present results, we propose that this paradoxical effect in women with PMDD emerges upon stress exposure. This hypothesis is further supported by the observation that women with PMDD have an enhanced anxiety-potentiated startle specifically in the luteal phase [48]. Since the anxiety-potentiated startle is mediated by the bed nucleus of the stria terminalis, where the GABAA receptor is widely expressed [49] and is sensitive to allopregnanolone manipulations [50], it may be inferred that the observed effect is the paradoxical effect of allopregnanolone in PMDD observed during stress processing, although a direct effect is yet to be established.

Our study was significantly impacted by the COVID-19 pandemic, resulting in a smaller sample size than originally planned. Hence, we were only able to show time-course differences in HF-HRV (i.e., change from baseline and return to baseline) according to group, but not group differences at each timepoint. Furthermore, our analyses are unadjusted. Hence, our findings will need to be replicated in a larger, properly powered study for more definitive conclusions. Nonetheless, the present study recruited a sample of women without illicit or prescription drug use, and without current psychiatric disorders, thereby removing a number of potential confounding effects. Moreover, we analyzed allopregnanolone using ultra-performance liquid chromatography tandem mass spectrometry, which distinguishes compounds with similar structures better than the immunoassay technology [51,52,53].

In summary, women with PMDD demonstrate a poor autonomic flexibility to respond to a psychosocial stress challenge, reflected by a higher parasympathetic nervous system withdrawal. This poor adaptive response to an environmental stressor seems to reflect an anxiogenic effect of allopregnanolone in PMDD. Further research is needed to examine underlying cellular and neurocircuit substrates of this interaction. In a broader sense, a greater understanding of the effects of stress and hormones on heart function is a critical area of research given the marked cardiovascular disease mortality in women.

## Figures and Tables

**Table 1:** Demographic characteristics according to study groups.

Demographic Variable	Category	Diagnosis		*p* Value
		PMDD (n = 17)	Healthy (n = 18)	
Age		26.05 (5.21)	26.44 (5.30)	0.83
Race	White	6	8	0.95
	Black or African American	2	3	
	American Indian or Alaska Native	1	0	
	Asian	6	6	
	Native Hawaiian or Other Pacific Islander	0	0	
	More than one race	1	1	
	Unknown/Do not want to specify	1	0	
Ethnicity	Hispanic	4	4	0.60
	Non-Hispanic	13	12	
	Unknown/Do not want to specify	0	2	
Student Status	Yes	11	6	0.09
	No	6	12	
Marital Status	Single	14	17	0.40
	Married	2	1	
	Divorced	1	0	
	Widowed	0	0	
Income	Less than $20,000	8	10	0.53
	$20,000—$34,999	1	3	
	$35,000—$49,999	4	1	
	$50,000—$74,999	2	3	
	75,000 or more	2	1	
Age of Menarche		12.06 (1.56)	11.66 (1.72)	0.53
BMI *		25.33 (5.26)	23.86 (4.08)	0.36
BDI **		3.00 (2.29)	2.94 (3.33)	0.95

Continuous variables are summarized as means (standard deviations). Data for categorical variables are presented as Ns. * BMI = Body Mass Index. ** BDI = Beck’s Depression Inventory.

**Table 2:** HRV Changes Across TSST Timepoints According to Diagnosis.

PMDD					
Contrast	Estimate	SE	df	t. Ratio	*p* Value
Baseline vs. Instruction	0.673	0.224	99	3.001	0.0176 *
Baseline vs. TSST	0.8	0.224	99	3.568	0.0031 **
Baseline vs. Recovery	0.396	0.224	99	1.768	0.2949
Instruction vs. TSST	0.127	0.224	99	0.567	0.9417
Instruction vs. Recovery	−0.276	0.224	99	−1.233	0.6076
TSST vs. Recovery	−0.404	0.224	99	−1.799	0.2797
Healthy					
Baseline vs. Instruction	0.2	0.218	99	0.918	0.7954
Baseline vs. TSST	0.518	0.218	99	2.378	0.0879
Baseline vs. Recovery	−0.121	0.218	99	−0.556	0.9448
Instruction vs. TSST	0.318	0.218	99	1.461	0.465
Instruction vs. Recovery	−0.321	0.218	99	−1.473	0.4573
TSST vs. Recovery	−0.639	0.218	99	−2.934	0.0213 *

* *p* ≤ 0.05; ** *p* ≤ 0.01. All contrasts were adjusted by the Tuckey method for comparing a family of 4 estimates.

## Data Availability

The data presented in this study are available on request from the corresponding author. The data are not publicly available due to preserve scientific integrity of research methodology.

## Supplementary Materials

The following supporting information can be downloaded at: https://www.mdpi.com/article/10.3390/jcm12041553/s1, Table S1: HRV Time Comparisons for the Overall Participant Sample; Table S2: HRV Time Comparisons for the Overall Participant Sample; Table S3: Mean, SD, and SEM of HRV Values According to Groups and Timepoints; Table S4: HRV Changes Across TSST Timepoints According to Diagnosis in the Partial Sample; Table S5: Mean, SD, and SEM of Allopregnanolone Concentration (ng/mL) According to Groups and Timepoints in the full sample.

## References

[1] American Psychiatric Association (2013). Diagnostic and Statistical Manual of Mental Health Disorders.

[2] Halbreich U., Borenstein J., Pearlstein T., Kahn L.S. (2003). The prevalence, impairment, impact, and burden of premenstrual dysphoric disorder (PMS/PMDD). Psychoneuroendocrinology.

[3] Kessler R.C., Walters E.E. (1998). Epidemiology of DSM-III-R major depression and minor depression among adolescents and young adults in the National Comorbidity Survey. Depress. Anxiety.

[4] Brownley K.A., Girdler S.S., Stout A.L., McLeod M.N. (2013). Chromium Supplementation for Menstrual Cycle-Related Mood Symptoms. J. Diet. Suppl..

[5] Gehlert S., Song I.H., Chang C.-H., Hartlage S.A. (2008). The prevalence of premenstrual dysphoric disorder in a randomly selected group of urban and rural women. Psychol. Med..

[6] Yonkers K.A., Simoni M.K. (2018). Premenstrual disorders. Am. J. Obstet. Gynecol..

[7] Epperson C.N., Steiner M., Hartlage S.A., Eriksson E., Schmidt P.J., Jones I., Yonkers K.A., Eisenlohr-Moul T.A., Girdler S.S., Schmalenberger K.M. (2012). Premenstrual Dysphoric Disorder: Evidence for a New Category for DSM-5. Am. J. Psychiatry.

[8] Beddig T., Kuehner C. (2017). Current Aspects of Premenstrual Dysphoric Disorder—A Review. Psychother. Psychosom. Med. Psychol..

[9] Kleinstäuber M., Schmelzer K., Ditzen B., Andersson G., Hiller W., Weise C. (2016). Psychosocial Profile of Women with Premenstrual Syndrome and Healthy Controls: A Comparative Study. Int. J. Behav. Med..

[10] Perkonigg A., Yonkers K.A., Pfister H., Lieb R., Wittchen H.U. (2004). Risk factors for premenstrual dysphoric disorder in a community sample of young women: The role of traumatic events and posttraumatic stress disorder. J. Clin. Psychiatry.

[11] Beddig T., Reinhard I., Kuehner C. (2019). Stress, mood, and cortisol during daily life in women with Premenstrual Dysphoric Disorder (PMDD). Psychoneuroendocrinology.

[12] Benjamin E.J., Virani S.S., Callaway C.W., Chamberlain A.M., Chang A.R., Cheng S., Chiuve S.E., Cushman M., Delling F.N., Deo R. (2018). American Heart Association Council on Epidemiology and Prevention Statistics Committee and Stroke Statistics Subcommittee. Heart Disease and Stroke Statistics-2018 Update: A Report From the American Heart Association. Circulation.

[13] Balzarotti S., Biassoni F., Colombo B., Ciceri M.R. (2017). Cardiac vagal control as a marker of emotion regulation in healthy adults: A review. Biol. Psychol..

[14] Thayer J.F., Sternberg E. (2006). Beyond Heart Rate Variability: Vagal Regulation of Allostatic Systems. Ann. N. Y. Acad. Sci..

[15] Malik M. (1996). Heart rate variability: Standards of measurement, physiological interpretation and clinical use. Task Force of the European Society of Cardiology and the North American Society of Pacing and Electrophysiology. Circulation.

[16] Hamidovic A., Van Hedger K., Choi S.H., Flowers S., Wardle M., Childs E. (2020). Quantitative meta-analysis of heart rate variability finds reduced parasympathetic cardiac tone in women compared to men during laboratory-based social stress. Neurosci. Biobehav. Rev..

[17] Martinez P.E., Rubinow D.R., Nieman L.K., Koziol D.E., Morrow A.L., Schiller C.E., Cintron D., Thompson K.D., Khine K.K., Schmidt P.J. (2015). 5α-Reductase Inhibition Prevents the Luteal Phase Increase in Plasma Allopregnanolone Levels and Mitigates Symptoms in Women with Premenstrual Dysphoric Disorder. Neuropsychopharmacology.

[18] Hantsoo L., Epperson C.N. (2020). Allopregnanolone in premenstrual dysphoric disorder (PMDD): Evidence for dysregulated sensitivity to GABA-A receptor modulating neuroactive steroids across the menstrual cycle. Neurobiol. Stress.

[19] Neckel H., Quagliotto E., Casali K.R., Montano N., Lago P.D., Rasia-Filho A.A. (2012). Glutamate and GABA in the medial amygdala induce selective central sympathetic/parasympathetic cardiovascular responses. Can. J. Physiol. Pharmacol..

[20] Johnston G.A.R. (2014). Muscimol as an Ionotropic GABA Receptor Agonist. Neurochem. Res..

[21] Salomé N., Ngampramuan S., Nalivaiko E. (2007). Intra-amygdala injection of GABAA agonist, muscimol, reduces tachycardia and modifies cardiac sympatho-vagal balance during restraint stress in rats. Neuroscience.

[22] Endicott J., Nee J., Harrison W. (2006). Daily Record of Severity of Problems (DRSP): Reliability and validity. Arch. Women’s Ment. Health.

[23] Gingnell M., Bannbers E., Wikström J., Fredrikson M., Sundström-Poromaa I. (2013). Premenstrual dysphoric disorder and prefrontal reactivity during anticipation of emotional stimuli. Eur. Neuropsychopharmacol..

[24] Solís-Ortiz S., Corsi-Cabrera M. (2008). Sustained attention is favored by progesterone during early luteal phase and visuo-spatial memory by estrogens during ovulatory phase in young women. Psychoneuroendocrinology.

[25] Sohda S., Suzuki K., Igari I. (2017). Relationship Between the Menstrual Cycle and Timing of Ovulation Revealed by New Protocols: Analysis of Data from a Self-Tracking Health App. J. Med. Internet Res..

[26] Kirschbaum C., Pirke K.M., Hellhammer D.H. (1993). The ‘Trier Social Stress Test’—A tool for investigating psychobiological stress responses in a laboratory setting. Neuropsychobiology.

[27] Li H.J., Goff A., Rudzinskas S.A., Jung Y., Dubey N., Hoffman J., Hipolito D., Mazzu M., Rubinow D.R., Schmidt P.J. (2021). Altered estradiol-dependent cellular Ca^2+^ homeostasis and endoplasmic reticulum stress response in Premenstrual Dysphoric Disorder. Mol. Psychiatry.

[28] Dubey N., Hoffman J.F., Schuebel K., Yuan Q., Martinez P.E., Nieman L.K., Rubinow D.R., Schmidt P.J., Goldman D. (2017). The ESC/E(Z) complex, an effector of response to ovarian steroids, manifests an intrinsic difference in cells from women with premenstrual dysphoric disorder. Mol. Psychiatry.

[29] Schmidt P.J., Martinez P.E., Nieman L.K., Koziol D.E., Thompson K.D., Schenkel L., Wakim P.G., Rubinow D.R. (2017). Premenstrual Dysphoric Disorder Symptoms Following Ovarian Suppression: Triggered by Change in Ovarian Steroid Levels But Not Continuous Stable Levels. Am. J. Psychiatry.

[30] Marrocco J., Einhorn N.R., Petty G.H., Li H., Dubey N., Hoffman J., Berman K.F., Goldman D., Lee F.S., Schmidt P.J. (2018). Epigenetic intersection of BDNF Val66Met genotype with premenstrual dysphoric disorder transcriptome in a cross-species model of estradiol add-back. Mol. Psychiatry.

[31] Porges S.W., Bohrer R.E. (1990). The analysis of periodic processes in psychophysiological research. Principles of Psychophysiology: Physical, Social, and Inferential Elements.

[32] Beck A.T., Ward C., Mendelson M. (1961). Beck depression inventory (BDI). Arch. Gen. Psychiatry.

[33] Beck A.T., Epstein N. (1988). An inventory for measuring clinical anxiety: Psychometric properties. J. Consult. Clin. Psychol..

[34] R Core Team (2020). R: A Language and Environment for Statistical Computing.

[35] Hamidovic A., Soumare F., Naveed A., Davis J., Sun J., Dang N. (2022). Reduced Dehydroepiandrosterone-Sulfate Levels in the Mid-Luteal Subphase of the Menstrual Cycle: Implications to Women’s Health Research. Metabolites.

[36] Baker F.C., Colrain I.M., Trinder J. (2008). Reduced parasympathetic activity during sleep in the symptomatic phase of severe premenstrual syndrome. J. Psychosom. Res..

[37] de Zambotti M., Nicholas C.L., Colrain I.M., Trinder J.A., Baker F.C. (2013). Autonomic regulation across phases of the menstrual cycle and sleep stages in women with premenstrual syndrome and healthy controls. Psychoneuroendocrinology.

[38] Landén M., Wennerblom B., Tygesen H., Modigh K., Sörvik K., Ysander C., Ekman A., Nissbrandt H., Olsson M., Eriksson E. (2004). Heart rate variability in premenstrual dysphoric disorder. Psychoneuroendocrinology.

[39] Matsumoto T., Ushiroyama T., Kimura T., Hayashi T., Moritani T. (2007). Altered autonomic nervous system activity as a potential etiological factor of premenstrual syndrome and premenstrual dysphoric disorder. Biopsychosoc. Med..

[40] Mumford S.L., Schisterman E.F., Gaskins A.J., Pollack A.Z., Perkins N.J., Whitcomb B.W., Ye A., Wactawski-Wende J. (2011). Realignment and multiple imputation of longitudinal data: An application to menstrual cycle data. Paediatr. Périnat. Epidemiol..

[41] Meng Y., Chang L., Hou L., Zhou R. (2022). Menstrual attitude and social cognitive stress influence autonomic nervous system in women with premenstrual syndrome. Stress.

[42] Oda Y. (2005). Influences of premenstrual syndrome on daily psychological states and salivary cortisol level. Jpn. J. Psychol..

[43] Andreen L., Sundström-Poromaa I., Bixo M., Nyberg S., Bäckström T. (2006). Allopregnanolone concentration and mood—A bimodal association in postmenopausal women treated with oral progesterone. Psychopharmacology.

[44] Miczek K.A., Barry H. (1977). Effects of alcohol on attack and defensive-submissive reactions in rats. Psychopharmacology.

[45] DeBold J.F., Miczek K.A. (1985). Testosterone modulates the effects of ethanol on male mouse aggression. Psychopharmacology.

[46] Miczek K.A., Weerts E.M., Tornatzky W., DeBold J.F., Vatne T.M. (1992). Alcohol and “bursts” of aggressive behavior: Ethological analysis of individual differences in rats. Psychopharmacology.

[47] Winslow J.T., Miczek K.A. (1988). Androgen dependency of alcohol effects on aggressive behavior: A seasonal rhythm in high-ranking squirrel monkeys. Psychopharmacology.

[48] Hantsoo L., Epperson C.N. (2015). Premenstrual Dysphoric Disorder: Epidemiology and Treatment. Curr. Psychiatry Rep..

[49] Lee Y., Davis M. (1997). Role of the Hippocampus, the Bed Nucleus of the Stria Terminalis, and the Amygdala in the Excitatory Effect of Corticotropin-Releasing Hormone on the Acoustic Startle Reflex. J. Neurosci..

[50] Toufexis D.J., Davis C., Hammond A., Davis M. (2004). Progesterone attenuates corticotropin-releasing factor-enhanced but not fear-potentiated startle via the activity of its neuroactive metabolite, allopregnanolone. J. Neurosci..

[51] Taylor A.E., Keevil B., Huhtaniemi I.T. (2015). Mass spectrometry and immunoassay: How to measure steroid hormones today and tomorrow. Eur. J. Endocrinol..

[52] Ney L.J., Felmingham K.L., Nichols D. (2020). Reproducibility of saliva progesterone measured by immunoassay compared to liquid chromatography mass spectrometry. Anal. Biochem..

[53] Stanczyk F.Z., Clarke N.J. (2010). Advantages and challenges of mass spectrometry assays for steroid hormones. J. Steroid Biochem. Mol. Biol..

