# Supplementary material for: Allopregnanolone Is Associated with a Stress-Induced Reduction of Heart Rate Variability in Premenstrual Dysphoric Disorder
Source: J Clin Med. 2023 Feb 16;12(4):1553. doi: 10.3390/jcm12041553 (PMC9967763; doi:10.3390/jcm12041553)
Supplement: Supplementary file 1 [file jcm-12-01553-s001.zip › jcm-2207594-supplementary.pdf]

Journal of
Clinical Medicine

Supplementary Table S1. HRV Time Comparisons for the Overall Participant Sample.

Contrast	Estimate	SE	df	t.ratio	p.value
Baseline vs Instruction	0.436	0.156	99	2.792	0.031*
Baseline vs Speech	0.659	0.156	99	4.216	0.0003***
Baseline vs Recovery	0.138	0.156	99	0.881	0.81
Instruction vs Speech	0.223	0.156	99	1.424	0.48
Instruction vs Recovery	-0.299	0.156	99	-1.911	0.23
Speech vs Recovery	-0.521	0.156	99	-3.335	0.0065**

*p≤0.05; **p≤0.01; ***p≤0.001.

Supplementary Table S2. HRV Time Comparisons for the Partial Participant Sample.

Contrast	Estimate	SE	df	t.ratio	p.value
Baseline vs Instruction	0.43	0.17	81	2.48	0.07#
Baseline vs Speech	0.62	0.17	81	3.59	0.003**
Baseline vs Recovery	0.14	0.17	81	0.81	0.85
Instruction vs Speech	0.19	0.17	81	1.11	0.69
Instruction vs Recovery	-0.29	0.17	81	-1.68	0.34
Speech vs Recovery	-0.48	0.17	81	-2.79	0.03*

#marginal, *p≤0.05; **p≤0.01; ***p≤0.001; Please refer to Supplementary Table 1 for comparison of these results in the full sample.

Supplementary Table S3. Mean, SD, and SEM of HRV Values According to Groups and Timepoints.

Timepoint	Group	n	Mean	SD	SEM
Baseline	PMDD	17	6.60	1.05	0.25
Baseline	Healthy	18	6.57	1.16	0.27
Instruction	PMDD	17	5.92	1.10	0.27
Instruction	Healthy	18	6.37	0.90	0.21
Speech	PMDD	17	5.80	1.30	0.32
Speech	Healthy	18	6.05	0.95	0.22
Recovery	PMDD	17	6.20	1.10	0.27
Recovery	Healthy	18	6.69	0.81	0.19

Supplementary Table S4. HRV Changes Across TSST Timepoints According to Diagnosis in the Partial Sample.

**PMDD**					
Contrast	*Estimate*	*SE*	*df*	*t.ratio*	*p.value*
Baseline *vs* Instruction	0.70	0.26	81	2.72	0.04*
Baseline *vs* TSST	0.75	0.26	81	2.90	0.02*
Baseline *vs* Recovery	0.36	0.26	81	1.40	0.50
Instruction *vs* TSST	0.05	0.26	81	0.18	1.00
Instruction *vs* Recovery	-0.34	0.26	81	-1.31	0.56
TSST *vs* Recovery	-0.39	0.26	81	-1.49	0.45
Healthy					
Baseline *vs* Instruction	0.16	0.23	81	0.70	0.90
Baseline *vs* TSST	0.50	0.23	81	2.15	0.15
Baseline *vs* Recovery	-0.08	0.23	81	-0.35	0.99
Instruction *vs* TSST	0.34	0.23	81	1.46	0.47
Instruction *vs* Recovery	-0.24	0.23	81	-1.05	0.72
TSST *vs* Recovery	-0.58	0.23	81	-2.51	0.07#

#marginal, *p≤0.05; **p≤0.01; All contrasts were adjusted by the Tuckey method for comparing a family of 4 estimates; Please refer to Table 2 for comparison of these results in the full sample.

Supplementary Table S5. Mean, SD, and SEM of Allopregnanolone Concentration (ng/mL) Accord-ing to Groups and Timepoints in the full sample.

Timepoint	Group	n	Mean	SD	SEM
-20	PMDD	16	1.13	0.71	0.18
-20	Healthy	16	1.79	1.77	0.44
20	PMDD	14	1.22	0.56	0.15
20	Healthy	16	1.69	1.31	0.33
30	PMDD	14	1.12	0.55	0.15
30	Healthy	16	1.79	1.38	0.34
40	PMDD	15	1.17	0.49	0.13
40	Healthy	16	1.79	1.35	0.34
50	PMDD	15	1.24	0.70	0.18
50	Healthy	16	1.70	1.28	0.32
65	PMDD	15	1.22	0.65	0.17
65	Healthy	16	1.66	1.28	0.32
90	PMDD	14	1.10	0.56	0.15
90	Healthy	15	1.66	1.06	0.27
